# A regulation probability model-based meta-analysis of multiple transcriptomics data sets for cancer biomarker identification

**DOI:** 10.1186/s12859-017-1794-6

**Published:** 2017-08-23

**Authors:** Xin-Ping Xie, Yu-Feng Xie, Hong-Qiang Wang

**Affiliations:** 1 School of Mathematics and Physics, Anhui Jianzhu University, Hefei, Anhui 230022 China; 2 Cancer Hospital, CAS, Hefei, Anhui 230031 China; 3 MICB Lab., Hefei Institutes of Physical Science, CAS, Hefei, 230031 China

**Keywords:** Cancer, Transcriptomics data, Meta-analysis, Differential expression, Regulation probability

## Abstract

**Background:**

Large-scale accumulation of omics data poses a pressing challenge of integrative analysis of multiple data sets in bioinformatics. An open question of such integrative analysis is how to pinpoint consistent but subtle gene activity patterns across studies. Study heterogeneity needs to be addressed carefully for this goal.

**Results:**

This paper proposes a regulation probability model-based meta-analysis, *j*GRP, for identifying differentially expressed genes (DEGs). The method integrates multiple transcriptomics data sets in a gene regulatory space instead of in a gene expression space, which makes it easy to capture and manage data heterogeneity across studies from different laboratories or platforms. Specifically, we transform gene expression profiles into a united gene regulation profile across studies by mathematically defining two gene regulation events between two conditions and estimating their occurring probabilities in a sample. Finally, a novel differential expression statistic is established based on the gene regulation profiles, realizing accurate and flexible identification of DEGs in gene regulation space. We evaluated the proposed method on simulation data and real-world cancer datasets and showed the effectiveness and efficiency of *j*GRP in identifying DEGs identification in the context of meta-analysis.

**Conclusions:**

Data heterogeneity largely influences the performance of meta-analysis of DEGs identification. Existing different meta-analysis methods were revealed to exhibit very different degrees of sensitivity to study heterogeneity. The proposed method, *j*GRP, can be a standalone tool due to its united framework and controllable way to deal with study heterogeneity.

**Electronic supplementary material:**

The online version of this article (doi:10.1186/s12859-017-1794-6) contains supplementary material, which is available to authorized users.

## Background

High throughput biotechnology has become a routine tool in biological and biomedical research [1, 2]. Its extensive applications have been generating and accumulating a flood of omics data that bring unprecedented opportunity for elucidating cancer or other diseases at a molecular level [3–6]. For example, various types of omics data for nearly 10,000 tumor or normal samples have been released from the cancer genome atlas (TCGA) project. In the two famous public databases, Gene Expression Omnibus (GEO) and ArrayExpress, there are millions of assays generated in more than 30,000 studies world-wide available online [7, 8]. To reduce sample bias and increase statistical power, one needs to reuse the flood of omics data in a meta-analysis way, gaining deeper insights into the molecular pathology of cancer or other diseases [9]. How to implement efficient meta-analysis of these data sets poses a pressing challenge for computational biologists and bioinformaticans.

Meta-analysis of transcriptomic data needs to interrogate consistent but subtle gene activity patterns across studies. Currently, there exist three categories of meta-analysis methods used for DEGs identification: *p*-value-based, effect size-based and rank-based. These methods deal with non-specific variations at different levels of data. For example, in statistics, *p*-value methods are most intuitive and allow for standardization of topic-related associations from studies to the common scale of significance. However, the performance of the *p*-value methods is stringently conditional on the estimation model of *p*-values used in individual analysis [10, 11]. To improve the situation, Li and Tseng [10] proposed an adaptively weighted strategy (AW) for *p*-value combination. Recently, Li et al. [12] introduced multiple test procedure and established assumption-weighting statistics, including I2, I2&direction, and mean cor, pooled cor, which are expected to settle down the heterogeneity and capture the concordance between different studies. Unlike the *p*-value methods, the effect size methods rely on a *t*-statistic-like model and can directly model the effect sizes across different studies. There are two commonly used effect size models in meta-analysis of transcriptomics data: fixed-effect model (FEM) and random effect model (REM), whose difference mainly lies in whether ignoring between-study variations or not. Compared with the *p*-value methods, the effect size methods are more sensitive to data distribution and noise inherent in microarray data, leading to unreliable effect size estimates [13].

As a non-parametric method, rank-based methods rely on combining the fold-change ranks, rather than combining *p*-values as in the *p*-value methods or expression levels as in the effect size methods. Compared with the effect size models, the rank-based methods make fewer or no assumptions about data structures in modeling differential expression of genes and thus runs more robust and outlier-free in performing meta-analysis for screening DEGs [14, 15]. A representative rank-based method is the *Rankprod* method proposed by Hong et al. [13]. In *Rankprod*, multiple fold changes are computed from all possible pair-wise comparisons of samples in each data set, and the rank product for each gene is then carried out by ranking the resulting fold changes within each comparison. For significance analysis, *Rankprod* assesses the null distributions of the rank product in each data set by Permutation tests. Unfortunately, *Rankprod* only work well for data sets where two categories of differential genes with two opposite directions are involved, and is less sensitive to inconsistent patterns of differential expression across studies [12, 16]. Additionally, Wang et al. proposed a matrix decomposition-based strategy for meta-analysis of transcriptomics data, which improves meta-analysis by mining differential physiological signals hidden behind multiple data sets [17].

A main issue in gene expression meta-analysis is how to deal with the study heterogeneity across data sets. The heterogeneity possibly comes from three sources: 1) Experimental environments. Gene expression datasets were often produced using different platforms and different processing facilities. Such kind of heterogeneity is often referred to as cross-lab/platform heterogeneity or batch effect [18]; 2) Incorrect gene annotations as technique mistakes, which occur when aligning target sequences or probes [19]; 3) Biological variability including various sub-subtypes of cancer or minor biological differences (e.g. age, gender or ethnicity). These heterogeneities could deteriorate identifying DEGs in meta-analysis if they are not addressed properly. Dealing with these heterogeneities should be simultaneously removing the non-specific heterogeneity and accommodating the minor biological ones properly. We previously proposed a regulation probability-based statistic for identifying DEGs in a single experiment, referred to as GRP [20]. The GRP model estimates the probabilities of two regulation events occurring between sample groups and allows to capture and control data noise or the intra-class heterogeneity. We here extend the model to deal with study heterogeneity in the context of meta-analysis of multiple data sets. Briefly speaking, the proposed method, joint GRP (*j*GRP), maps gene expression data across studies to a regulatory space and then measures expression difference in the regulatory space. In the resulted gene regulation profile, study heterogeneity can be efficiently captured and controlled by a regulation confidence parameter. We evaluated the proposed methods on both simulation data and real-world transcriptomic data sets, and experimental results demonstrate the superior performance of *j*GRP in gene expression meta-analysis for DEGs identification.

## Methods

The main idea of the proposed method is to integrate multiple expression data sets at the level of regulation rather than at the level of expression. More specifically, we produce a united gene regulation profile across studies from independent gene expression profiles and measure differential expression by characterizing the regulation property of genes between two conditions. Biologically, two opposite regulation events possibly occur in tumor relative to normal tissue for a given gene: up-regulation (*U*) and down-regulation (*D*). The former means that a gene expresses higher in tumor than does in normal tissue, while the latter means that a gene expresses lower in tumor than does in normal tissue. Let *P*(*U*) and *P*(*D*) represent the estimates of the two events’ probabilities, a regulation-based differential expression statistic can be defined as

1 $$ jGRP=P(U)-P(D) $$

The statistic *j*GRP∈[−1,1] reflects how likely the gene is regulated, whose positive value implies an up-regulation event occurring while whose negative value implies a down-regulation event occurring. A gene with a positive *j*GRP is potentially an onco-gene while the one with a negative *j*GRP is potentially a tumor suppressor. *P*(*U*) and *P*(*D*) need to be estimated in a gene regulation space. So, we first map gene expression profiles from microarrays or RNA-seq technology into a regulatory space, and the resulting gene regulation profiles can be used to estimate the two regulation probabilities, statistically.

### Mapping gene expression data to gene regulatory space

Suppose *T* studies each with two sample classes: tumor and normal tissue. For all the studies, we divide the total sample space into two subspaces: tumor subspace *S*_1_ and normal tissue subspace *S*_2_. For a given gene, we assume three regulation statuses in a sample: up-regulated one denoted by 1, down-regulated one denoted by −1, and non-regulated one denoted by 0. Considering a study *s* consisting of *n* tumor samples and *m* normal samples and a gene *g* whose expression levels in the tumor and normal tissue samples are *Y*_1_ = {*a*_11_, *a*_12_, …, *a*_1*n*_} and *Y*_2_ = {*a*_21_, *a*_22_, …, *a*_2*m*_} respectively, we can map the expression levels of gene *g* into a regulatory space as follows:

1) For the *i*th tumor sample with expression level *a*_1*i*_, its regulatory status can be determined as

2 $$ {r}_{1i}=\left\{\begin{array}{cc}\hfill 1\hfill & \hfill {l}_i\ge \tau \hfill \\ {}\hfill -1\hfill & \hfill 1-{l}_i>\tau \hfill \\ {}\hfill 0\hfill & \hfill others\hfill \end{array}\right. $$

where ${l}_i=\sum_{k=1}^mI\left({a}_{1i}\ge {a}_{2k}\right)/m$ represents the proportion of normal samples with an expression value not lower than *a*_1*i*_, and 0.5 ≤ *τ* ≤ 1 is a constant, referred to as regulation confidence cutoff, which controls the reliability of the inferred status. *I*(·) is an indicator whose value is one if the condition is true and zero else.

2) For the *i*th normal sample with expression level *a*_2*i*_, its regulatory status can be determined as

3 $$ {r}_{2i}=\left\{\begin{array}{cc}\hfill 1\hfill & \hfill {r}_i\ge \tau \hfill \\ {}\hfill -1\hfill & \hfill 1-{r}_i>\tau \hfill \\ {}\hfill 0\hfill & \hfill others\hfill \end{array}\right. $$

where ${r}_i=\sum_{k=1}^nI\left({a}_{2i}\le {a}_{1k}\right)/n$ represents the proportion of tumor samples with expression values not lower than *a*_2*i*_.

Combining Eqs.(2) and (3), the regulation profile of gene *g* in study *s* can be formulated as

4 $$ {R}_s={\left[-1,0,1\right]}^{m+n} $$

and then the united regulation profile across the *T* studies as

5 $$ R=\left[{R}_1,{R}_2,\cdots, {R}_T\right] $$

### Statistical estimation of *jGRP* statistic

Given the two sample subspaces *S*_1_ and *S*_2_, we estimate the two regulation events’ probabilities based on the regulatory statuses using the total probability theorem as follows:

6 $$ P(U)=P\left({Y}_1\right)P\left(U|{Y}_1\right)+P\left({Y}_2\right)P\left(U|{Y}_2\right) $$

and

7 $$ P(D)=P\left({Y}_1\right)P\left(D|{Y}_1\right)+P\left({Y}_2\right)P\left(D|{Y}_2\right) $$

where the prior probabilities of cancer and normal samples, *P*(*Y*_1_) and P(*Y*_2_), can be assessed as the proportions of cancer and normal samples in all the *T* studies respectively, and the rest four conditional probabilities can be assessed as the proportions of samples with up/down-regulated statuses in the corresponding subspace. Then, the statistic *j*GRP can be derived as

8 $$ jGRP=\frac{s_u-{s}_d}{n+m} $$

where *s*_*u*_ and *s*_*d*_ are the numbers of samples in which gene *g* is in up-regulated and down-regulated statues, respectively. Note that the summation (*S*) of *P*(*U*) and *P*(*D*) could vary around 1 depending on *τ*: *S* will be larger than one if *τ* ≤ 0.5 and be smaller than one else.

### Significance analysis of *j*GRP

We design a permutation test procedure for the significance analysis of *j*GRP. In the procedure, the labels of all samples across studies are randomly permuted *B* = 1000 times, and thus *B* permuted *j*GRPs can be obtained by running the *j*GRP procedure on the permutated data. The *B* permuted *j*GRPs provide an approximate to the null distribution of *j*GRP statistic, and so the significance level of an observed *j*GRP can be estimated as

9 $$ p\hbox{-} value=\frac{\sum_{i=1}^BI\left(\left|{jGRP}_i\right|\ge \left| jGRP\right|\right)}{B} $$

where *j*GRP*i*, *i* = 1,2,…,*B* represents the *i*th permuted *j*GRP from the permutation experiment.

## Results

### Evaluation on simulation data

#### Simulation data generation

Generally, study heterogeneity could come from: (i) Difference in the fraction of studies that show significantly differential expression in all the studies; (ii) Difference in different expression directions across studies. Accordingly, we generated two types of simulation data, simulation-I and II, which focus on the two aspects of heterogeneity respectively, by revising the procedure in [21].

Assume *T* = 10 studies each consisting of tumor and normal tissue groups of sizes randomly sampling from 4 to 15 and totally *G* = 10,000 genes to be considered. For simulation-I where DEGs are homogeneously differentially expressed, we simulated five categories of DEGs: differentially expressed in ten, eight, six, four and two studies, respectively. All the categories each were supposed to contain 500 genes, and the rest genes (7500) were assumed to be non-differential in any of the studies. For simulation-II, we assumed DEGs to be differentially expressed in different directions in different studies and considered two groups of categories of differential expression: The first group has differential expression in all ten studies, which consists of three categories: 1) differentially expressed in the same direction in all ten studies; 2) differentially expressed in seven of ten studies in one direction but in the rest (three) in the other direction; 3) differentially expressed in five of ten studies in one direction but in the rest (five) in the other direction. The second group have differential expression in six out of ten studies and consists of three categories: 1) differentially expressed in all six studies in the same direction; 2) differentially expressed in four of six studies in one direction, but in the rest (two) in the other direction; 3) differentially expressed in half studies in one direction, but in another half (three) in the other direction. Each of the six categories was assumed to contain 500 genes, and the rest genes (7000) were assumed to be non-differential in any of the studies. Tables 1 and 2 summarizes the details of the configuration of these simulation data.

**Table 1:** Differential expression settings of Simulation data-I/Simulation data-II

Category No.	Number of differential expression studies	Differential expression direction
1	10/10	Same/Same
2	8/10	Same/7:3
3	6/10	Same/5:5
4	4/6	Same/Same
5	2/6	Same/4:2
6	0/6	Same/3:3

**Table 2:** Top 20 KEGG pathways enriched in the DEG list of *j*GRP(τ = 0.7)

Term	*P*-value	BH-adjusted *p*-value
hsa04610:Complement and coagulation cascades	1.55E-07	4.61E-05
hsa04110:Cell cycle	4.60E-07	6.85E-05
hsa05150:Staphylococcus aureus infection	4.69E-07	4.66E-05
hsa05200:Pathways in cancer	7.69E-07	5.73E-05
hsa01130:Biosynthesis of antibiotics	1.28E-05	7.62E-04
hsa05222:Small cell lung cancer	4.42E-05	0.002192532
hsa05166:HTLV-I infection	4.90E-05	0.002081948
hsa04512:ECM-receptor interaction	8.49E-05	0.003157108
hsa04510:Focal adhesion	1.53E-04	0.005064416
hsa04640:Hematopoietic cell lineage	2.60E-04	0.007713087
hsa04514:Cell adhesion molecules (CAMs)	3.22E-04	0.008693226
hsa05133:Pertussis	3.93E-04	0.009705856
hsa04115:p53 signaling pathway	4.52E-04	0.01031831
hsa04668:TNF signaling pathway	6.53E-04	0.013813372
hsa05416:Viral myocarditis	6.59E-04	0.01300695
hsa05144:Malaria	7.11E-04	0.013154554
hsa05202:Transcriptional misregulation in cancer	7.72E-04	0.013454985
hsa05323:Rheumatoid arthritis	0.00129	0.021136644
hsa00051:Fructose and mannose metabolism	0.001356	0.021051205
hsa00480:Glutathione metabolism	0.00145	0.021393467

To synthesize the expression level of genes, we assume that the expression of each gene follows a normal distribution in each group and each study, i.e., the expression level *x*_*gsic*_ of a gene *g* in sample *i* of group *c* in study *s* was randomly sampled from $N\left({\mu}_{gsc},{\sigma}_{study}^2\right)$_._ Specifically, for the normal tissue group, the mean of expression was designed as *μ*_*gs*0_ = *μ* + *α*_*g*_ + *β*_*s*_ + (*αβ*)_*gs*_, where *μ* represents a constant background expression,${\alpha}_g\sim N\left(0,{\sigma}_{gene}^2\right)$ represents the gene bias, ${\beta}_s\sim N\left(0,{\sigma}_{study}^2\right)$ represents the study bias, and ${\left(\alpha \beta \right)}_{gs}\sim N\left(0,{\sigma}_{\mathrm{int}}^2\right)$ represents the gene-study interaction. For the tumor group, the mean of expression was *μ*_*gs*1_ = *μ*_*gs*0_for non-differential genes and *μ*_*gs*1_ = *μ*_*gs*0_ + *δ* + *υ*_*g*_ + *ε*_*gs*_for differential genes, where *δ* is the pooled mean expression difference,${\upsilon}_g\sim N\left(0,{\sigma}_{diff}^2\right)$ is the gene bias of the expression difference, and ${\varepsilon}_{gs}\sim N\left(0,{\sigma}_{derr}^2\right)$ is the gene-study interaction of the expression difference. We used two sets of the parameters $\left(\mu, {\sigma}_{gene}^2,{\sigma}_{study}^2,{\sigma}_{\mathrm{int}}^2,{\sigma}_{err}^2,\delta, {\sigma}_{diff}^2,{\sigma}_{derr}^2\right)$: A) (5,1.25, 0.49,0.25,0.16,0.8,0.0016,0.256) and B) (5,6.25,0.49,0.25,0.16,0.8,0.0016,0.256). Compared with A, B increases only the gene effect but retain other effects for investigating the influence of gene effect. In summary, four data scenarios were synthesized: Simulation I with parameter setting A (Simulation-IA) or parameter setting B (Simulation-IB), Simulation II with parameter setting A (Simulation-IIA) or parameter setting B (Simulation-IIB). For each data scenario, twenty data sets were randomly generated in the experiment and average results over them were used for algorithm evaluation.

#### Simulation data analysis

**Fig. 1:**
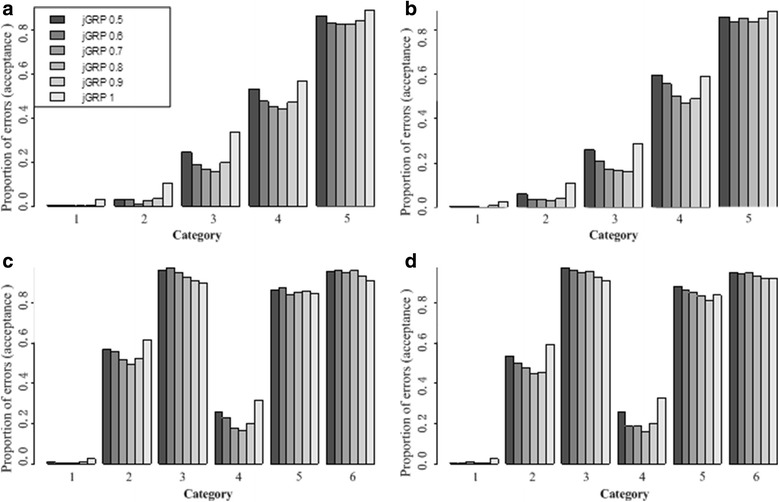
Proportions of errors (acceptance) of *j*GRPs in different categories of DEGs on four simulation data sets, simulation-IA (**a**), simulation-IB (**b**), and simulation-IIA (**c**), simulation-IIB (**d**)

Considering the importance of the regulation confidence cutoff parameter τ to the performance of *j*GRP, we varied τ = 0.5,0.6,0.7,0.8,0.9,1 and repeatedly applied *j*GRP to analyze the simulation data. To control false positive rates (FPR), the resulted *p*-values were corrected using the Benjamini-Hochberg (BH) procedure [22, 23]. Figure 1 summarizes the proportions of errors (acceptance) in each category of genes at an ad hoc BH-adjusted-*p*-value cutoff of 0.05 in the four data scenarios. From this figure, it can be found that, generally, too large or too small values of τ led to large errors, irrespective of any of the four data scenarios, as expected. The parameter τ directly controls the regulation confidence and captures the variation of differential expression across studies. Theoretically, too small *τ* can not filter out noise or non-specific heterogeneity such that DEGs will be recognized in a low confidence, leading to spurious DEGs, while too large τ means a too stringent control of study heterogeneity such that intra-class biological heterogeneity per se is excluded, missing true DEGs with complex patterns of differential expression. Relative to Simulation-IA, Simulation-IB have an increased gene effect, which led to slightly larger τ (around 0.8), at which the errors reach to the lowest, than that for simulation-IB (around 0.7) as shown in Fig. 1a-b. Similar results were observed between the two scenarios of Simulation-II, as shown in Fig. 1c-d.

Results also revealed that the error proportion gradually increases from Category 1 to 5 in both data scenarios of Simulation-I, as shown in Fig. 1a-b. This is consistent with the increasing heterogeneity of differential expression from Category 1 to 5. Similar phenomena were observed for Simulation-II (Fig. 1c-d). In Simulation-II, genes could be differentially expressed in different directions across studies, which produces additional heterogeneity for DEGs identification. Specifically, the heterogeneity increases from Category 1 to 3 and from Category 4 to 6. From Fig. 1c-d, we can clearly see that the error proportion gradually increases in a corresponding way across these categories, irrespective of Simulation-IIA or Simulation-IB. In summary, these results show that the proposed method can deal with various types of data heterogeneity across studies in a controllable way.

**Fig. 2:**
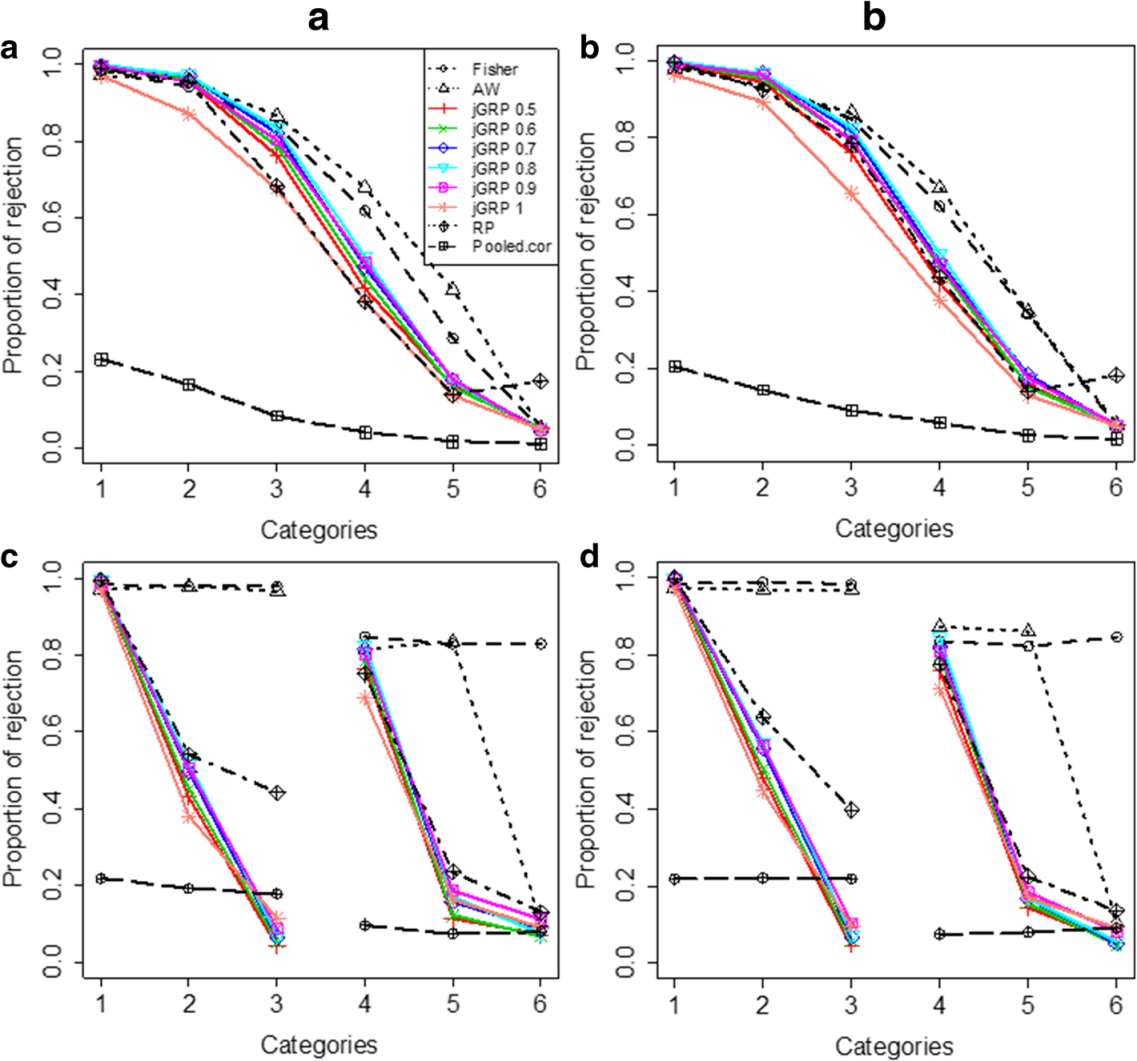
Comparison of the rejection proportions of jGRPs with those of previous methods on four simulation data sets, simulation-IA (**a**), simulation-IB (**b**), and simulation-IIA (**c**), simulation-IIB (**d**)

For comparison evaluation, we also applied previous methods, Fisher’s [24], AW [10], RankProd (RP) [25] and pooled cor [21], to analyze the simulation data. Two R packages, MetaDE and RankProd, were called to implement the two previous methods, AW and RP, respectively. For AW, the modt model was set (as default) to calculate the *p*-values for individual study and the fudge parameter to be the median variability estimator. Figure 2 compares the proportions of rejection (DEGs called) by *j*GRP at a BH-adjusted-*p*-value cutoff of 0.05 with those by the four previous method in the four data scenarios. As described above, study heterogeneity gradually increases from Categories 1 to 5 in the two scenarios of Simulation-I and from Categories 1(4) to 3(6) in the two scenarios of Simulation-II. It is expected that a reasonable method should be sensitive to the change of heterogeneity and have the proportions of rejection gradually drop as the heterogeneity increases across the categories in all the four data scenarios accordingly. From Fig. 2, we can clearly see that although *j*GRPs as well as the previous methods all are sensitive to the change of heterogeneity, they have different degrees of sensitivity in different simulation scenarios. Generally, the *p*-value-based methods led to the two extremes among these methods: Fisher’s and AW are least sensitive, while pooled cor is most sensitive. Especially, pooled cor seems too stringent to miss some DEGs that are even consistently differentially expressed across all the ten studies (Category 1) in all the four data scenarios. Lying in between the two extremes, *j*GRPs seems to be reasonably sensitive with a mild result in all the four data scenarios, and the sensitivity changes with the regulation confidence parameter in a controllable way: the larger or smaller the parameter the more sensitive *j*GRP. Results also reveals that RP is less sensitive to inconsistent expression patterns (Fig. 2c-d), which is consistent with the observations in [12]. In summary, *j*GRP shows a superior power of dealing with various types of study heterogeneity.

### Application to real microarray expression data

Considering that lung cancer is one of the most malignant tumors worldwide, we collected three real microarray lung adenocarcinoma (LUAD) cancer datasets from the GEO database: Landi’s data (GSE10072) [26], Selamat’s data (GSE32863) [27], and Su’s data (GSE7670) [28], in which all samples were divided into lung adenocarcinoma and normal (NTL). The Landi’s data consist of the expression levels of ~13,000 probes in total107 (58 LUAD and 49 NTL) samples; The Selamat’s data consist of the expression levels of ~25,000 probes in total 117 (58 LUAD and 59 NTL) samples; The Su’s data consist of the expression levels of ~13,000 probes in 54 (27 paired LUAD/NTL) samples. During generating these datasets, different microarray platforms were used to measure gene expression levels in parallel: Illumina Human WG-6 v3.0 Expression BeadChips for Landi’s data, HG-U133A Affymetrix chips for Selamat’s data, and Affymetrix Human Genome U133A array for Su’s data, which complicated data heterogeneity across these studies. We preprocessed the three datasets according to the following procedure: Averaging the intensities of multiple probes matching a same Entrez ID as the expression levels of the corresponding gene, and filtering out non-specific or noise genes by a CV filter (setting the CV cutoff as 0.05) [29]. As a result, the expression levels of 4728 common genes were used for meta-analysis for detecting LUAD-related DEGs.

**Fig. 3:**
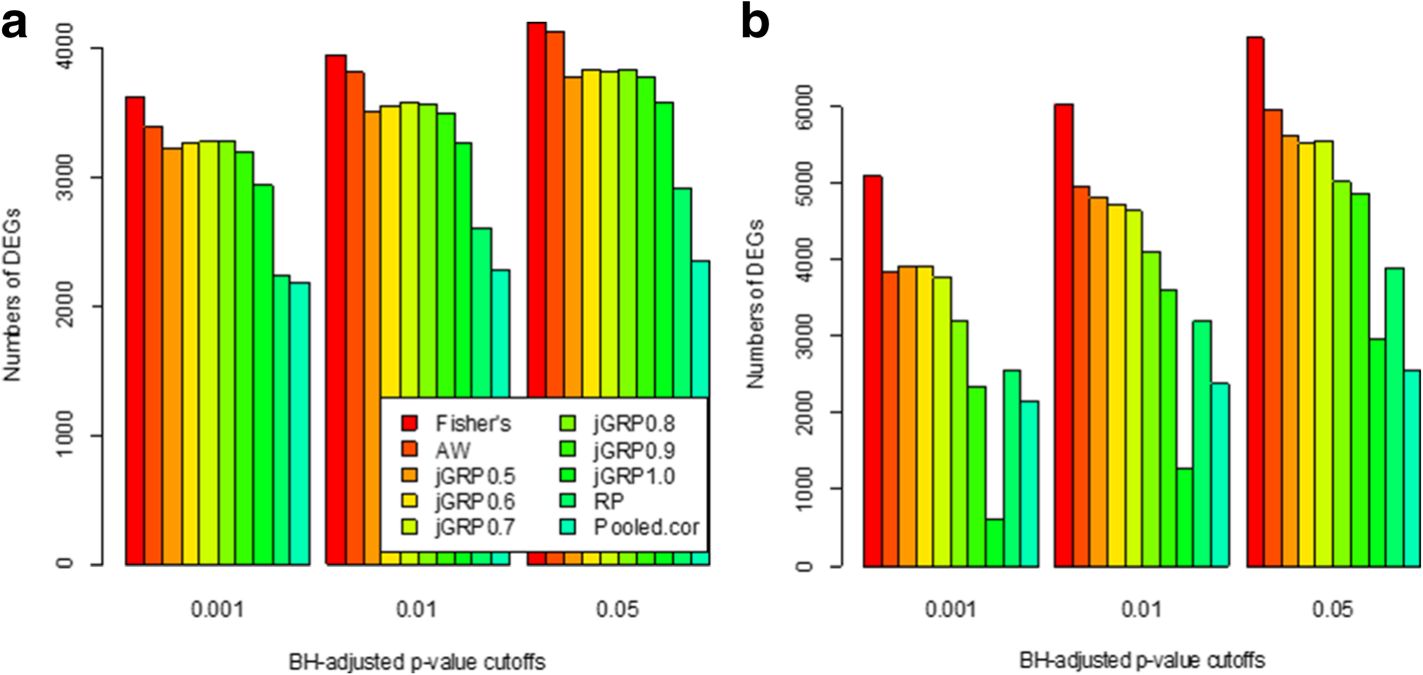
Comparison of numbers of DEGs identified by jGRPs and four previous methods, Fisher’s, AW, RP and pooled cor methods at BH-adjusted *p*-value cutoffs of 0.001, 0.01 and 0.05 for the three LUAD microarray data sets (**a**) and the two hepatocellular carcinoma RNA-seq data sets (**b**)

We applied *j*GRPs with varying *τ* = 05,0.6,0.7,0.8,0.9 and 1 to analyze the three data sets simultaneously. To control false positive rates (FPRs), the resulting *p*-values for each gene were corrected using Benjamini-Hochberg (BH) procedure [22, 23]. For comparison, four previous methods, Fisher’s [24], AW [10], RP [25] and Pooled cor [21], were also applied to re-analyze these data sets. Figure 3a shows the numbers of DEGs by *j*GRP and the previous methods at three BH-corrected *p*-value cutoffs of 0.001,0.01 and 0.05. From this figure, it can be clearly seen that our *j*GRP methods obtained a moderate result between the two previous methods, which is consistent with those on the simulation data above. Furthermore, for *j*GRPs, varying *τ* resulted in a similar changing pattern of the number of identified DEGs to those for the simulation data above, and *τ =* 0.7 obtained the largest and seemly more reasonable number of DEGs.

Results show that 3281 genes were called significantly differentially expressed between normal and LUAD tissues by *j*GRP (τ = 0.7) at an ad hoc BH-adjusted *p*-value cutoff of 0.001. Literature survey shows that many of these DEGs were previously reported to be related to lung cancer. For example, the gene with the largest value of *j*GRP (1), EPAS1, plays important roles in cancer progression and has been widely reported to be over-expressed in non-small cell lung cancer (NSCLC) tissues as a significant marker for poor prognosis [30, 31]. Other researchers have evidenced that in murine models of lung cancer, high expression levels of EPAS1 relate to tumor of large size, invasion and angiogenesis [32, 33].

One unique feature of *j*GRP is to automatically label DEGs with up-regulation or down-regulation in cancer. As a result, the 3281 DEGs were further divided by *j*GRP into two categories with different regulatory directions: 1655 (Additional file 1: Table S1) were with a negative *j*GRP statistic meaning a down-regulation in LUAD tissues relative to normal tissues, and 1626 (Additional file 1: Table S2) with a positive *j*GRP statistic meaning an up-regulation in LUAD. Among the 1655 down-regulated genes, many have been previously reported to be lowly expressed in lung tumor. For example, gene MTRR, which was missed by all the four previous method, Fisher’s, AW, RP and Pooled.cor, at an ad hoc BH-adjusted *p*-value cutoff of 0.001, was found with *j*GRP = −0.36 (*p*-value < 3 × 10^−5^, BH-corrected *p*-value < 5 × 10^−5^) to significantly down-regulated in LUAD. For this gene, Aksoy-Sagirli et al. [34] recently reported that its single-nucreotide polymorphism, MTRR 66 A > G, is significantly associated with lung cancer risk. Another gene, FAM107A with a large value of *j*GRP = −0.99 (*p*-value < 10^–16,^ BH-corrected *p*-value < 10^−16^), also named DRR1 and TU3A, is the member A of the family with sequence similarity 107, localized in chromosomal region 3p21.1 and ~10 kb long. Biologically, the protein that FAM107A encodes is involved in cell cycle regulation via apoptosis induction. It has been evidenced that FAM107A is frequently lost in various types of cancer, including ovarian cancer, cell carcinoma (RCC), prostate cancer and lung cancer cell lines [35, 36]. Recently, Pastuszak-Lewandoska et al. [37] observed that FAM107A was dramatically down-regulated in NSCLC samples relative to in tumor adjacent normal tissues. Gene TCF21 with a large value of *j*GRP (*j*GRP = −0.99, *p*-value < 10^−16^, BH-corrected *p*-value < 10^−16^), which encodes a transcription factor of the basic helix-loop-helix family, was extensively observed as tumor suppressor to under-express in human malignancies. Especially, Wang et al. [17] reported that the underrepresentation of TCF21 in LUAD tissues may be driven by its hypermethylation. The epigenetic inactivation in lung cancer was experimentally observed by Smith et al. [38] using restriction landmark genomic scanning. Recently, Shivapurkar et al. [39] adopted DNA sequencing technique to narrow down the sequence of TCF21 and pinpointed a short CpG-rich segment in the CpG island within exon 1 that is predominantly methylated in lung cancer cell lines but unmethylated in normal epithelial cells of lung. The short segment may account for the TCF21 expression abnormality in lung cancer. A more evidence reported by Richards et al. is that the association between hypermethylation and under-expression of TCF21 is specific to tumor tissues and occurs very frequently in various types of non-small cell lung cancer (NSCLC), even in the early-stage of NSCLC [40]. Taken together, these evidences confirm the down-regulation pattern of TCF21 in LUAD and suggest that it may be driven by its hypermethylation.

Among the 1626 up-regulated genes, many have also been previously reported to be under-expressed in lung cancer. For example, gene STRN3, which was missed by RP and Pooled.cor at an ad hoc BH-adjusted *p*-value cutoff of 0.001, was found to be up-regulated in LUAD with *j*GRP = 0.32 (*p*-value < 5 × 10^−4^, BH-corrected *p*-value < 7 × 10^−4^). As a single marker, STRN3 efficiently distinguished 100 NSCLC patients from 147 control subjects with a sensitivity of 84% and a specifity of 81%, and was included into a membrane array-based assay for non-invasive diagnosis of patients with NSCLC [41]. Another gene COL11A1 with a large value of *j*GRP (*j*GRP = 0.97, *p*-value < 10^−16^, BH-corrected *p*-value < 10^−16^) has been previously reported to take part as a minor fibrillar collagen in cell proliferation, migration and the tumorigenesis of many human malignancies. For example, Shen et al. [42] experimentally observed that the gene was significantly up-regulated in recurrent NSCLC tissues and in NSCLC with lymph node metastasis. It has been revealed that Smad signaling functionally mediates the overexpression of COL11A1 in NSCLC cells during the cell proliferation, migration and invasion of NSCLC cell lines in vitro. COL11A1 can act as a biomarker for clinical diagnosis of metastatic NSCLC [42]. For gene HMGA1 (*j*GRP = 0.97, *p*-value < 10^−16^, BH-corrected *p*-value < 10^−16^), the two previous methods, pooled cor and Fisher’s, ranked it at 183th and after 1000, respectively. Biologically, HMGA1 encodes a protein that is functionally associated with chromatin, which is involved in the metastatic progression of cancer cells. Previous studies reported that HMGA1 is widely over-expressed in a variety of aggressive tumors, suggesting that HMGA1 may act as a convictive biomarker for NSCLC prognostic prediction [43]. Especially, using immunohistochemistry, Zhang et al. [44] found that increased protein levels of HMGA1 are positively correlated with the status of clinical stage, classification of T, N and M, and differentiated degree in NSCLC.

To further assess the DEGs identified by different methods, we also perfomed pathway enrichment analysis using the commonly used online DAVID tool (http://david.abcc.ncifcrf.gov/home.jsp). As a result, DAVID reported 42, 57, 53, 40, 20 KEGG pathways (Additional file 1: Table S3-S7) significantly enriched in the DEG lists of *j*GRP (τ = 0.7) and four previous methods, Fisher’s, AW, RP and Pooled cor, at an ad hoc *p*-value cutoff of 0.05, respectively. Compared with the previous methods, *j*GRP gave higher ranks to pathways that are related to cancer progression, including cell cycle (Rank 2) comprised of a series of cellular events that leads to the division and duplication of DNA (DNA replication) of a cell, and small cell lung cancer (Rank 6), as shown in Table 2. Especially, the Complement and coagulation cascades pathway ranked at 1 was recently reported to dysfunction in lung cancer [45, 46]. *j*GRP also called another two lung cancer-related pathways, NF-kappa B signaling pathway and PI3K-Akt signaling pathway, but pooled cor did not. In NF-kappa B signaling pathway, nuclear factor-κB (NFκB) is a family of transcription factors that regulate the expression of genes that are involved in cell proliferation, differentiation and inflammatory responses. It has been widely evidenced that activating FκB can induce tumorigenesis of normal cells [47–49].

### Application to RNA-seq expression data

We also evaluated the performance of the proposed method on RNA-seq expression data. Hepatocellular carcinoma (HCC) is the third leading cause of cancer-related deaths. Two HCC RNA-seq data sets were collected from the GEO database: Liu’s data (GSE77314) [50] and Dong’s data (GSE77509) [51], both of which were measured using Illumina Hiseq 2000, and jointly analyzed them for identifying HCC biomarkers. The former consists of mRNA profiles of 50 paired normal and HCC samples, and the latter consists of mRNA profiles of 40 matched HCC patients and adjacent normal tissues. For quality control, we preprocessed the two datasets by averaging the FPKM values with a same Entrez ID as the expression levels of the corresponding gene and filtering out non-specific or noise genes based on a CV filter [29]. As a result, two HCC expression data sets containing 4945 common genes were jointly analyzed for identifying HCC-related DEGs.

Similar to the three LUAD microarray data sets, we applied *j*GRPs with varying *τ* = 05,0.6,0.7,0.8,0.9, 1 and the four previous methods, Fisher’s [24], AW [10], RP [25] and Pooled cor [21], to jointly analyze the two RNA-seq data sets, respectively, and corrected *p*-values using Benjamini-Hochberg (BH) procedure [22, 23] for controlling false positive rates. Figure 3b shows the numbers of DEGs called by *j*GRPs and the previous methods at three BH-corrected *p*-value cutoffs of 0.001,0.01 and 0.05. Similar to Fig. 3a, b reveals that most of *j*GRPs obtained an intermediate result between those by the previous methods, Fisher’s, AW and Pooled cor, for the HCC RNA-seq data. Among the *j*GRPs, the one with τ = 0.6, which is smaller than 0.7 for the LUAD data sets above, led to a more reasonable result, implying that it is more heterogeneous across the two HCC data sets than that across the three LUAD data sets. The high heterogeneity may be the reason for the unusually large numbers of DEGs by RP which is less sensitive to inconsistent patterns of expression [12].

Totally, there were 1724 genes called significantly differential expressed between normal and HCC tissues by *j*GRP (τ = 0.6) at a BH-adjusted *p*-value cutoff of 0.001. Among them, 1206 (Additional file 1: Table S8) were with a negative *j*GRP statistic, i.e., a down-regulation in HCC tissues relative to normal tissue, and 518 (Additional file 1: Table S9) with a positive *j*GRP statistic, i.e., an up-regulation in HCC. The imbalance of up- and down-regulated genes informed a higher degree of heterogeneity across the two HCC data sets compared with that across the three LUAD data sets (1655 down-regulated DEGs and 1626 up-regulated DEGs), which is in concordance with the unusually larger numbers of DEGs by RP. Then, we examined the biological functions of the two sets of DEGs. Literature survey shows that many of them have been previously reported to relate to HCC or cancer. For example, one of down-regulated DEGs, Nat2, with *j*GRP = −1, *p*-value < 10^–16^ and BH-corrected *p*-value < 10^–16^, can both activate and deactivate arylamine and hydrazine drugs and carcinogens. Some polymorphisms in Nat2 have been previously reported to increase the risk of HCC and drug toxicity [52, 53]. Recently, it has been widely observed that Nat2 are consistently and stably down-regulated in more than three hundred HCC patients [54]. One of up-regulated DEGs, CDC20, with *j*GRP = 1, *p*-value < 10^–16^, and BH-corrected *p*-value < 10^–16^, biologically acts as a regulatory unit in cell cycle that interacts with several proteins at multiple points of cell cycle. Li et al. [55] reported that high expression of CDC20 is associated with development and progression of hepatocellular carcinoma. Recently, CDC20 has been suggested to be a potential novel cancer therapeutic target [56]. We also conducted pathway analysis using the DAVID tool on the 1724 DEGs. As a result, 39 KEGG pathways (Additional file 1: Table S10) were called to be significantly enriched in the DEG list at an ad hoc *p*-value cutoff of 0.05, many of which were previously found to be involved in tumorigenesis, e.g., cell cycle and p53 signaling pathway. Especially, a new pathway, i.e., Bile secretion pathway, was found to be significantly enriched and relate to HCC (*p*-value = 2.5 × 10^–8^), which though needs to be further investigated by pathologists. Biologically, Bile is a vital secretion, which is essential in digesting and absorbing fats and fat-soluble vitamins in the small intestine. There are two mechanisms that influence Bile secretion: membrane transport systems in hepatocytes and cholangiocytes and the structural and functional integrity of the biliary tree. The dysfunction of the two mechanisms may cause the signaling abnormality of the Bile secretion pathway in HCC.

## Discussion

The central problem in transcriptomics data meta-analysis is how to deal with study heterogeneity. The heterogeneity complicates the distribution of gene expression and thus hinders accurately pinpointing the concordance of differential expression across studies. Two intuitive alternative approaches for data integration could be 1) Directly use the information contained in several data-sets; and 2) Cluster higher/lower expressed genes in each data-set and then zoom in on the interesting genes. However, they both ignore or inappropriately deal with the gene expression heterogeneity problem between studies. Currently, most methods for meta-analysis of differential expression directly operate in gene expression space, which are based on either *p*-values, ranks, or hierarchical *t*-statistic models. The proposed method, *j*GRP, at the first time establishes a universal and flexible integrative framework that operates in gene regulation space instead of in gene expression space, in which individual samples from different sources are more compatible. The regulation profile for a sample is derived from its expression profile based on probabilistic theory, where biological variability and noise inherent in gene expression data are modeled efficiently in combination with an adjustable parameter. It is also intuitive and simple to implement and easy to use in practice. We expect that this work can promote a research interest in borrowing gene regulation knowledge for integrative identification of DEGs.

The regulation confidence cutoff parameter τ reflects a tradeoff between regulation confidence and noise accommodation and is of importance to the performance of *j*GRP. How to properly choose the parameter is still an open question. The choice should be conditional on the study heterogeneity at hand. Here, we would like to recommend 0.7 as default for the parameter for simplicity or to try different values among 0.5 and 1 and then choose a proper value, depending on a particular data condition.

## Conclusions

We have presented a novel transcriptomic data meta-analysis method, *j*GRP, for identifying differentially expressed genes. The method integrates multiple gene expression data sets in a gene regulatory space instead of in the original gene expression space, which makes it easy to relieve the data heterogeneity between cross-lab or cross-platform studies. To produce the regulatory space, two gene regulation events between two conditions were mathematically defined, whose occurring probabilities were estimated using the total probabilistic theorem. Based on the resulting gene regulation profiles, a novel statistic, *j*GRP, was established to measure the differential expression of a gene in the regulatory space. *j*GRP introduces a parameter (*τ*) for users to conveniently adjust to fit into various levels of study heterogeneity in practice. Compared with existing methods, *j*GRP provides a united and flexible framework for DEGs identification in a meta-analysis context and is intuitive and simple to implement in practice, which can be a standalone tool due to the superior power of dealing with study heterogeneity. We evaluated the proposed method on simulation data and real-world microarray and RNA-seq gene expression data sets, and experimental results demonstrate the effectiveness and efficiency of *j*GRP for DEGs identification in gene expression data meta-analysis. Future work will be focused on guidelines for the choice of the regulation confidence cutoff parameter and biological verification of the new DEGs identified in the real applications.

## Additional file

Additional file 1: Table S1. List of 1655 genes with a negative *j*GRP statistic meaning a down-regulation in LUAD tissues relative to normal tissues on the three LUAD data sets. **Table S2.** List of 1626 genes with a positive *j*GRP statistic meaning a up-regulation in LUAD tissues relative to normal tissues on the three LUAD data sets. **Table S3.** List of 42 KEGG pathways significantly enriched in the DEG lists of *j*GRP (τ = 0.7) by DAVID. **Table S4.** List of 57 KEGG pathways significantly enriched in the DEG lists of Fisher’s by DAVID. **Table S5.** List of 53 KEGG pathways significantly enriched in the DEG lists of AW by DAVID. **Table S6.** List of 40 KEGG pathways significantly enriched in the DEG lists of RP by DAVID. **Table S7.** List of 20 KEGG pathways significantly enriched in the DEG lists of Pooled cor by DAVID. (RAR 259 kb)
